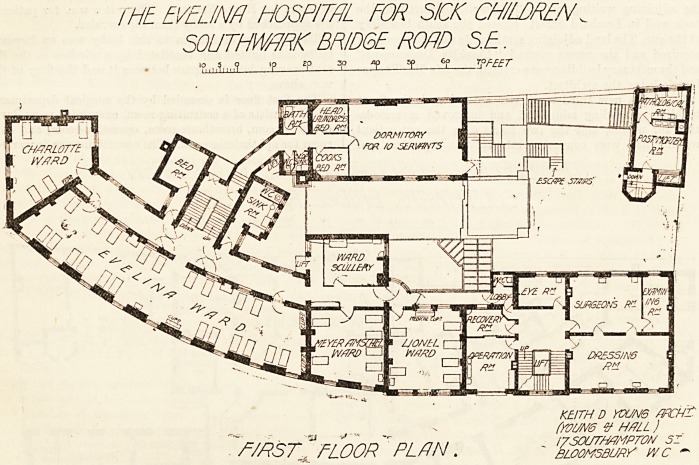# The Evelina Hospital for Sick Children

**Published:** 1908-05-23

**Authors:** 


### HOSPITAL ADMINISTRATION.

### CONSTRUCTION AND ECONOMICS.

### THE EVELINA HOSPITAL FOR SICK CHILDREN.

THIS hospital was erected in 1869 from designs by Mr. V. Low. The original building extended southwards only as far as the wall which now separates the dispensary from the adjoining waiting-room. This wall, produced to the back wall in Lombard Street, formed the south boundary of the site. The land adjoining southwards was subsequently acquired and the rooms marked "Casualty Department" and the mortuary building were erected, the late Mr. Thomas Harris being the architect.

In 1902 a new third story was erected over the front building, providing additional and improved accommodation for nurses, and the two floors over the out-patient waiting hall were converted into servants' quarters and room, room for the dental surgeon, and the dispensary waiting-room. The dispensary is in the old building and now occupies the space formerly taken up by a sitting-room and bedroom for House Surgeon. There is a w.c. for patients' use approached by a lobby from the corridor.

Though cross-ventilation to this lobby was an impossibility it is lighted and ventilated by a window in the flat roof opening into the space between it and the floor of the w.c. above.

The first floor is occupied by the surgical department, which consists of a consulting-room, examining-room, large dressing-room, anæsthetic-room, operation-room, and dark room for ophthalmic work. The operation-room is warmed

THE EVELINA HOSPITAL FOR SICK CHILDREN
SOUTHWARK BRIDGE ROAD S.E.

KEITH D. YOUNG ARCHT
(YOUNG & HALL)
17 SOUTHAMPTON ST
BLOOMSBURY W.C

GROUND FLOOR PLAN

a complete isolation department. This work was carried out by Messrs. Young and Hall.

The very inadequate and defective accommodation for out-patient work had for some time been occupying the attention of the Committee of Management, and after fruitless efforts to obtain a more extended site it was decided to make the best of the land already in the possession of the hospital and remodel the out-patient department in the best way possible in the circumstances. The comparatively small area available rendered it absolutely necessary that the department should be spread over two stories; an arrangement undesirable where it can be avoided, but the inconvenience of which is very much lessened by the help of an electric lift.

The new wing contains on the basement floor the electric department, mattress store, drug store, and a new scullery adjoining the kitchen. On the ground floor is the physician's consulting-room with examining-room and a large dressingby means of a hot-water radiator, behind which fresh air is admitted from the outside, being passed through a jute screen which intercepts the coarser particles of floating matter. An extraction shaft carried up the whole height of the building and furnished with an electric fan provides for the efficient movement of air. The room itself and the anæsthetic-room adjoining is lined as to its walls and ceiling with glass tiles and has a floor of vitreous mosaic. The whole of the rooms on ground and first floors and the corridors throughout are paved with vitreous mosaic, and the consulting and other rooms and the staircase have a high dado of vitreous tiles, the upper parts of the walls being painted with enamel paint. The vitreous tiles, though they present a rougher surface than ordinary enamel or glass tiles are perfectly washable, and have the advantage of a much greater range of colour than glass tiles, while being considerably less costly than enamel tiles.

On the second floor are bedrooms for the matron, assistant matron and one sister, a sitting-room for the assistant matron, bathroom, w.c. and housemaids' closet, and ward for sick nurses. On the third floor are six bedrooms for nurses and sisters, similar bathroom and sanitary offices and a lecture-room. The roof over the new wing is flat and paved with asphalt, and is used for an airing ground for patients and nurses.

The old Out-Patient Department has been much altered. The one consulting-room has become casualty-room, and the two small rooms adjoining, formerly operation-room and eye room, have been thrown together and are now used as casualty dressing-room. The partitions forming the old dressing-room and House Surgeon's room have been swept away and the space thrown into the waiting hall. The old dispensary has been formed into two bathrooms, and new w.c.'s for patients fixed in a space formerly the only place available for isolating a child with infectious disease pending removal by ambulance. A room in the basement, approached by the outside staircase leading out of the lobby by the casualty dressing-room is now available as an isolation-room.

Among other minor alterations may be mentioned the formation of a lobby, waiting-room, porter's office and telephone room out of the formerly wasted space of the entrance hall; the provision of an additional bedroom for the resident staff by dividing up the room next entrance hall; the provision of a bathroom for the resident staff, and, finally, a new boiler which provides an ample supply of hot water for all purposes to take the place of three inadequate and wasteful boilers.

A comparison of the plans of the hospital as it stands to-day with those published in "Hospitals and Asylums of the World," showing it as it was when it was opened is interesting, not only as evidence of the development of hospital requirements, but as showing how a poorly contrived plan on an inadequate and inappropriate site can be improved within certain somewhat narrow limits. It shows also how, by careful administration and vigilant care on the part of the medical and nursing staff, the defects inherent to such a building can be rendered of no effect.

The recent alterations and additions have been designed and carried through by Mr. Keith D. Young (Young and Hall).

## Figures and Tables

**Figure f1:**
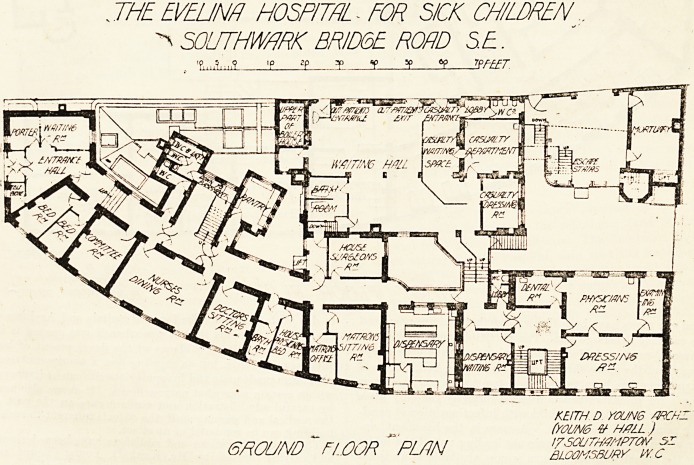


**Figure f2:**